# Supplementary material for: Lower Survival and Increased Circulating Suppressor Cells in Patients with Relapsed/Refractory Diffuse Large B-Cell Lymphoma with Deficit of Vitamin D Levels Using R-GDP Plus Lenalidomide (R2-GDP): Results from the R2-GDP-GOTEL Trial
Source: Cancers (Basel). 2021 Sep 15;13(18):4622. doi: 10.3390/cancers13184622 (PMC8471526; doi:10.3390/cancers13184622)
Supplement: Supplementary file 1 [file cancers-13-04622-s001.zip › cancers-1364506-SI.pdf]

Supplementary Material: Lower Survival and Increased Circulating Suppressor Cells in Patients with Relapsed/Refractory Diffuse Large B-Cell Lymphoma with Deficit of Vitamin D Levels Using R-GDP Plus Lenalidomide (R2-GDP): Results from the R2-GDP-GOTEL Trial

Carlos Jiménez-Cortegana, Pilar Sánchez-Martínez, Natalia Palazón-Carrión, Esteban Nogales-Fernández, Fernando Henao-Carrasco, Alejandro Martín García-Sancho, Antonio Rueda, Mariano Provencio, Luis de la Cruz-Merino and Víctor Sánchez-Margalet

Table S1. Spearman correlations between cell populations and basal vitamin D levels in the R2-GDP-GOTEL patients before and after treatment.

**Determination vs. Vitamin D**	**Basal (*n* = 63)**		**End of induction (*n* = 24)**	
M-MDSCs	***rS***	0.131	*rS*	−0.576
	P value	0.371	*P value*	0.016*
G-MDSCs	*rS*	−0.315	*rS*	−0.064
	P value	0.015*	*P value*	0.776
Total MDSCs	*rS*	0.211	*rS*	−0.308
	P value	0.108	*P value*	0.153
Regulatory T cells	*rS*	−0.274	*rS*	−0.521
	P value	0.037*	*P value*	0.013*
CD4+PD−1+OX40− T cells	*rS*	0.102	*rS*	−0.454
	P value	0.440	*P value*	0.030*
CD8+PD−1+OX40− T cells	*rS*	0.108	*rS*	−0.169
	P value	0.441	*P value*	0.431
Total PD−1+OX40− T cells	*rS*	0.133	*rS*	−0.322
	P value	0.310	*P value*	0.116
CD4+CTLA−4+OX40− T cells	*rS*	0.052	*rS*	−0.708
	P value	0.691	*P value*	<0.001***
CD8+CTLA−4+OX40− T cells	*rS*	−0.192	*rS*	−0.431
	P value	0.146	*P value*	0.036*
Total CTLA−4+OX40− T cells	*rS*	−0.098	*rS*	−0.735
	P value	0.456	*P value*	<0.001***

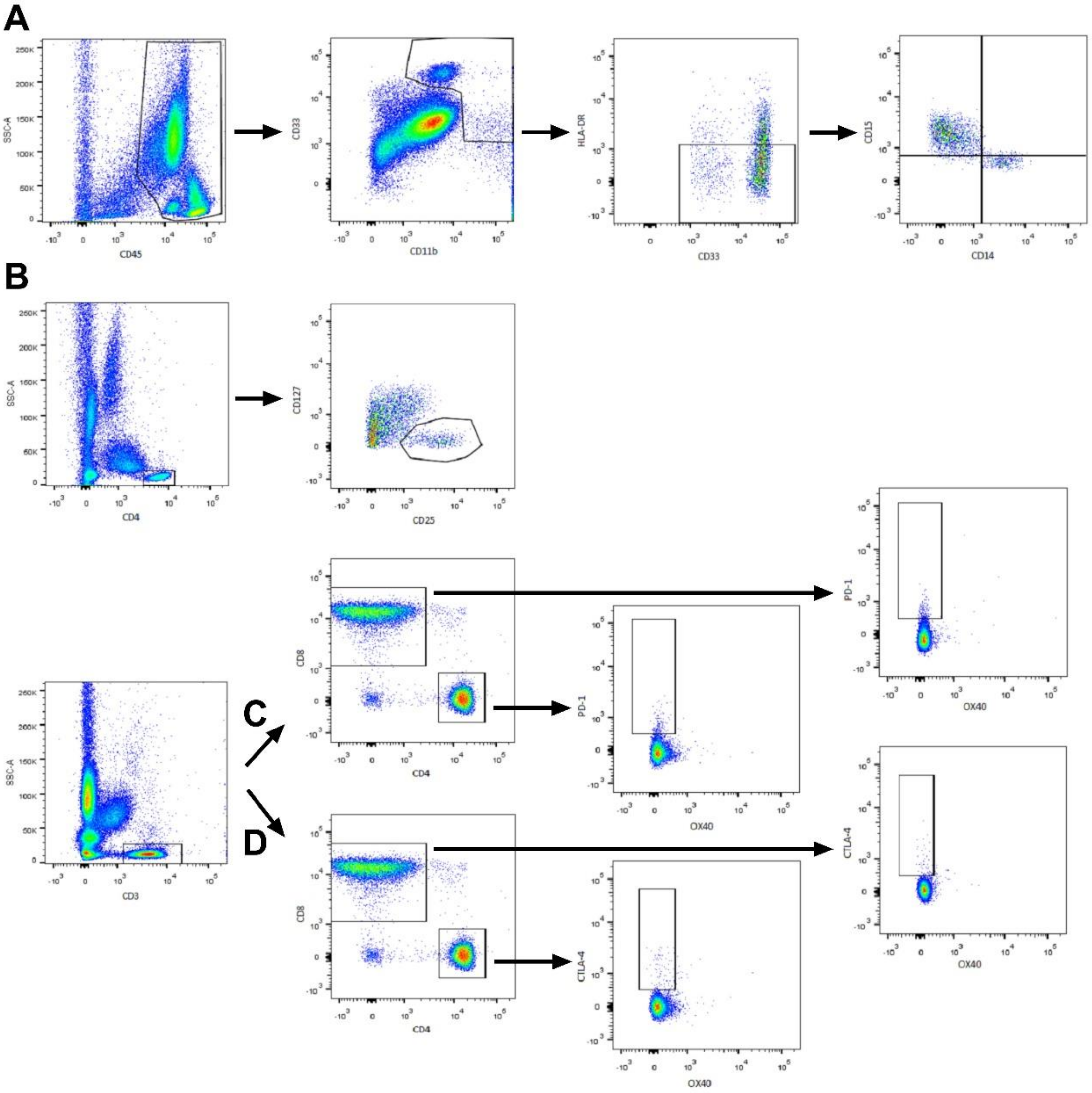

Figure S1. Flow cytometry gates for immune response suppressor cells. (**A**) Myeloid-derived suppressor cells**;** (**B**) Regulatory T cells; (**C**) PD−1+OX40− T cells; (**D**) CTLA−4+OX40− T cells.

Publisher's Note: MDPI stays neutral with regard to jurisdictional claims in published maps and institutional affiliations.

© 2021 by the authors. Licensee MDPI, Basel, Switzerland. This article is an open access article distributed under the terms and conditions of the Creative Commons Attribution (CC BY) license (http://creativecommons.org/licenses/by/4.0/).
